# Lifestyle intervention as a treatment for obesity among school-age-children in Celaya, Guanajuato: An experimental study

**DOI:** 10.5195/cajgh.2013.21

**Published:** 2013-03-29

**Authors:** Nicolas Padilla-Raygoza, Rosalina Diaz-Guerrero, Ma. Laura Ruiz-Paloalto

**Affiliations:** 1 Department of Nursing and Obstetrics, Health Sciences and Engineering Division, Celaya-Salvatierra Campus, University of Guanajuato; 2 Department of Clinical Nursing, Health Sciences and Engineering division, Celaya-Salvatierra Campus, University of Guanajuato; 3 Division of Health Sciences and Engineering, Campus Celaya Salvatierra, University of Guanajuato

**Keywords:** hypertension, physical activity, meals

## Abstract

**Introduction:**

Obesity is a risk factor in chronic diseases, and its frequency among children in Mexico is increasing.

**Objective:**

To determine the effect of lifestyle intervention as a treatment for obesity in school-age-children from Celaya, Mexico.

**Methodology:**

For this experimental study, four schools were randomly selected. Children and parents participated voluntarily and signed consent forms. Two schools were chosen as the experimental group and the other two formed the control group. Age, gender, weight, height, BMI and blood pressure were recorded for each participant.

**Intervention:**

Children and parents were asked to walk in their schools for 30 minutes a day Monday through Friday and to attend 8 instructional sessions over a period of four months dedicated to the selection and preparation of meals.

**Statistical Analysis:**

The OR and 95% CI were calculated to determine the effect of the intervention; a Z-test for two proportions for overweight and obesity in the control and experimental groups were carried out for comparison.

**Results:**

157 children were included in the experimental group and 144 in the control group. To compare the proportions of the overweight and the obese between the groups, a Z-test = −0.36 (p-value 0.72) were obtained showing no effect of the intervention in lifestyle; OR =1.09, 95% CI (0.67, 1.77). It was adjusted according to the attendance to the sessions resulting in an OR = 2.00, 95% CI (0.69, 5.77), demonstrating that not attending the sessions was a confounder.

**Conclusions:**

Intervention in lifestyle should be measured over a longer period of time in order to determine what effects it may have on changes in body mass index.

## Introduction

Obesity is a chronic condition that contributes to many diseases and affects more children and adolescents each day. One of the most important concerns is the emergence of type-II diabetes and metabolic syndrome at earlier ages.^1^

Obesity is the result of an imbalance between energy consumption and expenditure due to diets high in caloric density and low in fiber, as well as high consumption of sugary drinks accompanied by little or no physical activity.^2^

In Mexico, according to the National Surveys on Health and Nutrition, the frequency of obesity in male and female children and adolescents has increased from 18.6% in 1999 to 26% in 2006, The percentage of overweight children and adolescents has also increased from 12.9% in 1999 to 21.2% in 2006.^3^,^4^

In the United States, the Centers for Disease Control and Prevention (CDC) is also concerned with this issue. Their studies show that no state reported an obesity rate above 15% in children and adolescents in 1990, but in 1995 more than half the states reported an obesity rate between 15 and 19 %. By the year 2000, over 22 states had rates higher than 20%, and in 2005 17 states had rates above 25%.^5^

From 2003 to 2004, the National Health and Nutrition Examination Survey (NHANES) reported that 33.6% of children and adolescents had some degree of obesity; 17.1% were obese and 16.5% at risk of obesity.^6^

Early weaning, increased intake of processed foods with a high caloric density, a decrease in consumption of low caloric density foods (fruits and vegetables), urbanization, mechanization of transport and use of technology, a decrease in energy expenditure, less time spent on leisure activities, and decreased time and intensity in physical education and sports at school all contribute to obesity at an early age.^3^

An obese patient is 2.5 times more likely to develop coronary heart disease, 4 times more likely to develop hypertension, 3–4 times more likely to develop type-II diabetes and at 5 times higher risk of stroke than a person with a BMI in the normal range.^7^

The percentage of newly-enrolled elementary school students in Mexico who are obese has increased by a factor of three, suggesting that approximately 26% of Mexican children reach obesity before the onset of puberty.^3^

The effects of obesity and being overweight among children are numerous, leading to conditions such as: glucose intolerance, insulin resistance, type-II diabetes, hypertension, dyslipidemia, hepatic steatosis, sleep apnea, orthopedic problems, low self-esteem, negative body image and depression, discrimination, negative stereotypes, anorexia and bulimia, among others. These effects prove an overwhelming burden to the healthcare system.

Therefore, we must seek strategies to prevent or decrease the number of obese and overweight children.

The objective of this study was to apply an educational and physical activity intervention program to treat or prevent increased weight in order to lower the frequency of this syndrome in school age children from Celaya, Guanajuato, Mexico.

We developed the following hypothesis: Children in the experimental group will have a 10% decreased frequency of overweightness/obesity compared with the control group.

## Materials and Methods

The protocol was reviewed and approved by Bioethics Committee from the School of Nursing and Obstetrics of Celaya, University of Guanajuato.

### Type of study

This prospective, controlled, longitudinal study was carried out in public elementary schools from Celaya, Guanajuato, Mexico.

### Population

Male and female school-age-children, enrolled in Celaya public elementary schools incorporated to the Guanajuato Ministry of Education.

### Inclusion criteria

Children 6 to 13 years old, male or female enrolled in public elementary schools, whose parents accepted their participation in the study by signing the corresponding consent form.

### Exclusion criteria

Children with diseases that inhibited physical activity, children whose parents did not sign the consent form, and children over the age of 8 who did not consent.

### Sampling

Four schools were selected at random from the 168 existing public schools in Celaya in 2007. Two schools randomly formed the experimental group and the other two were the control group. At each school, parents were invited to an informative meeting where the objectives of the study were explained. Parents were asked to sign a consent form allowing their children to participate in the study. Children 8 or older were asked to sign an additional form demonstrating their own wish to participate. All participants answered questions in reference to their age, gender, residence, and socioeconomic level (SELI).^8^ Participants were weighed using the Medidata Serie MS ® digital scale. They wore only essential clothing and did not wear shoes. They stood on the scale looking forward while both weight and height appeared on the screen of the scale. Blood pressure was measured 3 times using a digital monitor. The average of the three readings was calculated. A correlation between measurements was established by measuring the blood pressure of 30 children using a digital and a mercurial monitor (adjusting the cuff to the age of the participant). The correlation was r= 0.79.

Body Mass Index (BMI) was calculated by dividing weight in kilograms by height (in centimeters) squared. The mothers of the children answered a questionnaire on food habits (SNUT)^9^ and a software was used to process the SNUT and obtained the daily average of calorie consumption.

All measurements were carried out at the beginning of the study and at sixteen weeks. The results found in the control and experimental groups were used to define the impact of the lifestyle intervention program.

### Intervention

It was decided that the lifestyle intervention program be administered at the schools to avoid contamination of the control group by the experimental group in a non-school environment.

The lifestyle intervention program in the experimental group had two phases:

### Phase 1

All school-age-children, whose parents agreed to participate in the study, had monitored 30 -minute walking sessions at school from Monday through Friday where attendance was checked. To maximize the participation in the walking sessions, they were carried out at the end of their school day (12:30 PM).

### Phase 2

8 Instructive sessions with the children’s mothers were carried out. In these sessions, mothers were taught to prepare and select healthy meals for their children. The sessions took place every two weeks throughout the 16 weeks of the study. Two nutritionists were in charge of the sessions. Table 1 shows the descriptive content of the sessions. Attendance was checked at each session.

### Follow-up

After sixteen weeks, anthropometric measurements and blood pressure were measured. The SNUT survey was then applied to both the experimental and control groups.

The control group did not receive any lifestyle intervention. However, after the study was over, a meeting was held with the mothers of the control group to instruct them on the advantages of physical activity and selection and preparation of healthy meals as preventive measures to avoid overweight/obesity. This was done in order to meet the bio-ethical principles of justice.

### Outcome measurements

“Overweight” was defined as a BMI between the 75th and 84th percentile in accordance with the CDC 2000 curves by age and gender”.^10^ “Obesity” was defined as a BMI in the 85th–96th percentile in accordance with the CDC 2000 curves, by age and gender”.^10^ “Severe obesity” was defined as a BMI above the 97th percentile in accordance with the CDC 2000 curves, by age and gender”.^10^ “Hypertension” was defined as pressure levels higher than the 95^th^ percentile in the blood pressure charts, by gender, age and height”.^11^

### Sample size

The expected proportion of overweight/obese was 26% in the control group and 16% in the experimental group. The minimum sample size with a 95% of precision and an 80% of power is 101 in each group. An increase to 150 should be considered due to conglomerate sampling (design factor 1.5) (EpiInfo 2000 version 1.1 CDC, Atlanta, GA, EUA).

### Statistical Analysis

Children were classified as overweight/obese, or appropriate weight. Another classification was hypertensive or with appropriate blood pressure. The proportion of overweight/obese in each group was compared with the Z-test and p-value to test the hypothesis. The same test was used to compare rates of hypertension between the experimental and control groups. To measure the effects of the lifestyle intervention program, the Odds Ratio (OR) and 95% Confidence Intervals (CI) between groups were calculated for overweight/obese and for hypertension. It was adjusted in accordance with the attendance to the walking sessions and the preparation and selection of meals sessions.

As a second step, the subjects were classified by status of obesity: low/adequate weight, overweight, obese, and severely obese. Proportions between the experimental and control group were compared with the Z-test for two proportions and p-value.

Using a Chi-squared test and p-value, tabulation was elaborated comparing the status of hypertension and the status of obesity to determine if there was a relationship between variables.

Mean differences in weight, height, BMI, systolic blood pressure, diastolic blood pressure, and daily calorie intake before and after the intervention were calculated with the Z-test and p-value.

In all cases, the p-value used to demonstrate statistical significance was 0.05. All statistical analysis was calculated using STATA 10.0® (Stata Corp, Texas, EUA).

## Results

400 parents from the 4 selected schools were invited to attend the informational meetings. Among these 400 parents, 297 (74.25%) permitted their children to participate in the study and signed the consent form. The experimental group was comprised of 157 children and the control group of 144.

Qualitative baseline characteristics in each group are shown in Table 2.

In terms of age, X^2^ = 1.16, df= 2, p = 0.6, point to no significant difference in the groups. In terms of gender, there was also no significant difference among groups with X^2^ = 2.68, df=1, p = 0.1. In terms of the socioeconomic level index, the Z-test for two proportions between height (SELI), was Z=0.03, p=0.97.

In the walking sessions, the mean of the absences of children was 2.71±1.25 and in the instructional sessions for mothers on selecting and preparing the children’s meals was 14±0.35. No participant had more than 20% absence in both activities.

Table 4 shows the quantitative baseline characteristics between the experimental and the control groups. The Z-test was calculated for two independent means. In terms of age, the average difference was Z = −0.47, p = 0.64; in terms of weight in kilograms, the average difference was 1.85 with Z = 1.24 and p = 0.2. Height was measured in meters and the difference was 0 with Z = 0 and p = 1.0. The differences in these variables were not statistically significant. On the other hand, when measuring systolic blood pressure in mm Hg, the difference was 4.96, with Z = 3.18, p = 0.001. The difference in diastolic blood pressure was 4.45 with Z = 3.45, p = 0.0006; in terms of BMI, the difference in the mean was 1.21 with Z = 2.25, p = 0.03. With regards to daily consumption of calories, the average difference was 465 with Z = 4.88, p = 0.0000. These differences were statistically significant.

Three children from the experimental group (1.9%) and two from the control group (1.4%) decided to drop out from the study.

To test the hypothesis of comparing the proportions of the overweight/obese between experimental and control groups, a Z-test resulting in −0.36 was calculated and p=0.72. This demonstrates that there is no significant statistical difference between both groups and that the lifestyle intervention program had no effect, with OR=1.09 and a 95% CI=0.67 to 1.77.

When adjusting the OR with absences to the walking and instructional sessions on selecting and preparing meals, the OR adjusted was 2.00 [CI 95% (0.69, 5.77)], showing that the attendance to the walking sessions and instructional sessions were factors of confusion.

Proportions of overweight/obese children, BMI, and hypertension were compared before and after the intervention program.

Statistically significant differences were found in the hypertension category in the experimental group, in the overweight/obesity category, and in the BMI categories (p<0.05).

In pre and post-intervention analysis of the quantitative variables, we calculated the differences between the first and the second measurements, the mean of the differences, the standard deviation, the paired t-test, and p-value.

In terms of weight, a significant statistical difference was found between the groups (p<0.05). For height, differences between the experimental and the control group were statistically significant as well (p<0.05). The same was obtained in the BMI (p<0.05), in systolic blood pressure (p<0.05), and in diastolic blood pressure (p<0.05) for both groups; and the same was obtained for the average of daily consumption of calories in the experimental and control group (p<0.05) (Table 6).

## Discussion

The sample of schools was obtained by random selection, which helps control for potential bias; however, the children participating were invited to partake. This introduces a possibility of bias since the parents that were interested in the study and accepted the invitation to participate probably had obese children. Evidence for this can be seen in the difference in baseline values between groups (Table 4).

3 subjects (1.9% of the group) from the experimental group and 2 (1.4% of the group) from control group decided to drop out of the study, but this did not affect the analysis of the data.

Results obtained from this study do not support the hypothesis that children in the experimental group will have a 10% decrease compared with the control group (Table 5).

The percentages of 64.94% in the experimental group and 66.90% in the control group were higher than those reported by the National Survey on Health and Nutrition in Mexico during 2006 of 26%^4^.

115 (73.25%) children in the experimental group were already overweight / obese before the lifestyle intervention program began. After the intervention program only 100 (64.94%) children were overweight / obese. Although this may not be statistically significant, there was a substantial decrease in this particular group. The control group started out with 84 (58.33%) children that were overweight / obese and after the 16 weeks it increased to 95 (66.90%) children. (Table 5).

It can also be observed that obese and overweight children in the experimental group lost weight after the lifestyle intervention program (p<0.05), and that the number of children with appropriate weight increased. This demonstrates, in a way, that our lifestyle intervention program was effective. In the control group, the number of students with appropriate weight decreased from 60 to 47 (p> 0.05) and the increase in overweight school children was statistically significant (p <0.05) (Table 5).

An important and unexpected finding was the frequency of hypertension, 12.74% in the experimental group and 2.78% in the control group, since in Mexico the hypertension rate is considered to be at 1% in children.^12^ In the experimental group, it was detected that after 4 months of the application of the intervention program, there were no children with hypertension in the experimental group and only 1 child with hypertension in the control group (Table 5). These results could be biased because in the first visit, the children were unfamiliar con observers and maybe they felt stress; this could decrease after 16 weeks. Steinberger et al. reported an association between obesity and hypertension in children and adolescents^13^. Physical activity at least 30 minutes, 3 times/week, reduces blood pressure in youth with mild essential hypertension.^14^

The intervention of physical activity and adequate preparation and selection of meals proved to be effective. The experimental group showed an average increase of 1.25 Kg, in weight; whereas the control group had an average increase of 2.75 Kg after the four months of the study. There was also a change in height after the follow-up time, but this may be due to the natural growth of the children. (Table 6).

Average difference in BMI was similar; 0.45 in the experimental group and 0.46 in the control group, (Table 6).

Systolic blood pressure in the experimental group had an average decrease of 5.05 mmHg after the lifestyle intervention program, while in the control group it increased 1.95 mmHg. There were also differences in the diastolic blood pressure; the average decrease in the experimental group was 3.21 mmHg; there was an average increase of 1.81 mmHg in the control group (Table 6). Even though the analyses performed before and after the intervention program were statistically significant for both groups, the results were less significant in the experimental group as compared to the control group. It must be pointed out that the experimental group showed positive differences in systolic and diastolic blood pressure, indicating the second measurements were lower than the first measures. In contrast, the control group had a negative difference, indicating that the second measurements were higher than the first (Table 6).

Average daily calorie intake decreased by 823 calories in the experimental group, while the control group increased their intake by 164 calories (Table 6). Even though in the analyses made before and after the intervention program for both groups were statistically significant, the experimental group showed positive differences for daily mean intake of calories, indicating that the second measurements were lower than the first measures. In contrast, the control group had a negative difference, indicating that the second measurements were higher than the first (Table 6).

Klesges et al. monitored a group of females for 2 years whom were following an intervention program designed to prevent obesity. The major foci of the program were: drinking more water, increasing the consumption of vegetables and fruits, and lowering the intake of sweetened beverages. They measured the BMI of the experimental and control groups and significant differences were not found.^15^ The intervention program in our study lasted four months. BMI was slightly modified in the experimental and control groups but the change was not statistically significant.

The World Health Organization recommends that changes in lifestyle (such as increased physical activity) is an important part in the prevention and treatment of hypertension and also helps in the treatment of obesity.^16^,^17^

## Conclusions

The null hypothesis could not be rejected because the differences in proportions of school children with in the experimental and control group were not statistically significant.

With physical activity intervention and changes in the selection and preparation of meals, the number of children with hypertension decreased.

Both increased physical activity and changes in food selection and preparation should be studied over a longer period of time because four months did not show sufficient effect on BMI. An important effect of the intervention was a decrease in blood pressure.

These results warrant further investigation of the effects of healthy lifestyles on changes in BMI in school children because of the far-reaching benefits that may come from improving the health of young people.

## Figures and Tables

**Table 1:** Descriptive chart of contents from instructional sessions with mothers to help to select and prepare meals, Celaya, Gto. 2009

Session	Topic	Objective	Teaching Strategy
1	Growth and development	To know the importance of foods in childhood	Lecture with slides
2	Nutrients needed for growth and development	To recognize which are the most important nutrients for growth and development in children	Lecture with slides
3	Foods groups	To familiarize mothers with the food groups for an appropriate nutrition.	Workshop style and collaborative group work.
4	Food portions	To recognize the appropriate food portions for each child.	Workshop style and collaborative group work.
5	Practical advice	To learn practical ways to get your child to eat healthy meals.	Lecture with slides
6	Myths and realities about feeding	To answer frequently asked questions about child nutrition.	Lecture with slides
7	Food preparation workshop I	To empower mothers in preparing nutritious meals.	Preparation and tasting of healthy meals
8	Food preparation workshop II	To empower mothers in preparing nutritious meals.	Preparation and tasting of healthy meals

**Table 2:** Qualitative baseline characteristics of both groups, Celaya,Gto., 2009 (n= 301)

Variables	Experimental group (n=157)		Control group (n=144)	
	n	%	n	%

Age group (years)				
6 – 8	78	49.68	61	42.36
9 – 11	67	42.68	75	52.08
12 – 14	12	7.64	8	5.56

Gender				
Male	90	57.32	68	47.22
Female	67	42.68	76	52.78

SELI				
Low	0	0	1	0.69
Regular	11	7.01	8	5.56
High	146	92.99	135	93.75

Source: questionnaires from study.

SELI= Socioeconomic level index

**Table 3:** Attendance to walking sessions and instructional sessions in to select and prepare meals in experimental group, Celaya, Gto, 2009 (n=154)

	n	%
Absences in walking sessions		
0	2	1.30
1	24	15.58
2	42	27.27
3	50	32.47
4	25	16.23
5	7	4.55
6	3	1.95
7	1	0.65

Absences to instructional sessions		
0	132	85.71
1	22	14.29

Source: Checklist of attendance

**Table 4:** Quantitative baseline characteristics of both groups, Celaya, Gto., 2009 (n=301)

Variables by group	Range	Mean ± SD

Age (in years)		
Experimental (n=157)	6 to 13	8.74 ± 1.93
Control (n=144)	6 to 13	8.84 ± 1.73

Systolic blood pressure (mmHg)		
Experimental (n=157)	80 to 180	115.06 ± 16.03
Control (n=144)	85 to 155	110.12 ± 10.68

Diastolic blood pressure (mmHg)		
Experimental (n=157)	42 to 125	75.24 ± 12.44
Control (n=144)	46 to 122	70.79 ± 9.59

Weight (Kg)		
Experimental (n=157)	17.850 to 84.950	37.28 ± 13.87
Control (n=144)	17.800 to 76.950	35.43 ± 12.79

Height (Mt)		
Experimental (n=157)	0.82 to 1.64	1.32 ± 0.13
Control (n=144)	1.06 to 1.61	1.32 ± 0.12

Body mass index (Kg/m^2^)		
Experimental (n=157)	13.52 to 42.24	20.88 ± 4.84
Control (n=144)	13.49 to 33.75	19.67 ± 4.47

Mean of daily calorie consumption		
Experimental (n=157)	1471.23 to 6909.62	2803.75 ± 859.52
Control (n=144)	755.05 to 6049.45	2338.29 ± 790.80

Source: Questionnaires of study, SNUT survey

SD=Standard deviation

**Table 5:** Comparison before and after intervention by overweight/obesity status and hypertension, Celaya, Gto, 2009 (n=301)

	Before			After	Difference of proportions	Z	p-value
	n	%	n	%			

Experimental							
Overweight/obesity	115	73.25	100	64.94	0.0831	1.59	0.11
Without overweight/obesity	42	26.75	54	35.06	−0.0831	−1.59	0.11
Control							
Overweight/obesity	84	58.33	95	66.90	−0.0857	−1.50	0.13
Without overweight/obesity	60	41.67	47	33.10	0.0857	1.50	0.13

Experimental							
Severe obesity	60	38.22	40	25.97	0.1225	2.31	0.02
Obesity	35	22.29	45	29.22	−0.0693	−1.40	0.16
Overweight	20	12.74	15	9.74	0.03	0.84	0.40
Without overweight	42	26.75	54	35.06	−0.0831	−1.59	0.11
Control							
Severe obesity	34	23.61	32	22.54	0.0107	0.21	0.83
Obesity	36	25.00	37	26.06	−0.0106	−0.21	0.83
Overweight	14	9.72	26	18.31	−0.0859	−2.09	0.04
Without overweight	60	41.67	47	33.10	0.0857	1.50	0.13

Experimental							
Hypertension	20	12.74	0	0	0.1274	4.58	0.0000
Without hypertension	137	87.26	154	100.0	−0.1274	−4.58	0.0000
Control							
Hypertension	4	2.78	1	0.70	0.0208	1.34	0.18
Without hypertension	140	97.22	141	99.30	−0.0208	−1.34	0.18

Source: questionnaires of the study

**Table 6:** Comparison of quantitative parameters pre/post-intervention, per group, Celaya, Gto, 2009 (n=301)

	Before	After	δ ± sd (IC95%)	t-paired	Df	p-value
	Mean ± s	Mean ± s				

Weight (Kg)						
Experimental	37.28±13.87	38.39±13.93	−1.25±1.23 (−1.45to −1.05)	−12.61	153	0.0000
Control	35.43±12.79	38.05±14.77	−2.75±5.89 (−3.73 to −1.77)	−5.56	141	0.0000

High (Mt)						
Experimental	1.32±0.13	1.35±0.14	−0.04±0.02 (−0.04 to −0.036)	−24.82	153	0.0000
Control	1.32±0.12	1.34±0.13	−0.02±0.06 (−0.04 to −0.037)	−23.83	141	0.0000

BMI						
Experimental	20.88±4.84	20.41±4.59	−0.46±0.96 (−0.61 to −0.31)	−5.95	153	0.000
Control	19.67±4.47	20.09±4.33	−0.45±1.65 (−0.72 to −0.18)	−3.25	141	0.001

SAT (mmHg)						
Experimental	115.06±16.03	109.97±8.69	5.05±14.85 (2.69 to 7.41)	4.22	153	0.0000
Control	110.12±10.68	112.14±8.25	−1.95±7.64 (−3.22 to −0.68)	−3.04	141	0.003

DAT (mm Hg)						
Experimental	75.24±12.44	71.86±6.24	3.21±11.88 (1.32 to 5.10)	3.35	153	0.001
Control	70.79±9.59	72.69±5.68	−1.81±8.96 (−3.30 to −0.32)	−2.41	141	0.017

MDIC						
Experimental	2803.75±859.52	1988.41±639.51	803.25±879.42 (663.25 to 943.25)	11.33	153	0.0000
Control	2338.29±790.80	2502.67±661.65	−164.16±522.91 (−250.91 to −77.41)	−3.74	141	0.0003

BMI= Body mass index δ± sd Mean of differences ± standard deviation

SAT= systolic blood pressure DAT= diastolic blood pressure MDIC= Mean daily intake of calories
